# Potential Hydrothermal-Humification of Vegetable Wastes by Steam Explosion and Structural Characteristics of Humified Fractions

**DOI:** 10.3390/molecules26133841

**Published:** 2021-06-24

**Authors:** Wenjie Sui, Shunqin Li, Xiaodan Zhou, Zishan Dou, Rui Liu, Tao Wu, Hongyu Jia, Guanhua Wang, Min Zhang

**Affiliations:** 1 State Key Laboratory of Food Nutrition and Safety, College of Food Science and Engineering, Tianjin University of Science & Technology, Tianjin 300457, China; wjsui@tust.edu.cn (W.S.); sqli18306891445@163.com (S.L.); dawnzhou5@163.com (X.Z.); xiaoweidanao@163.com (Z.D.); lr@tust.edu.cn (R.L.); wutao@tust.edu.cn (T.W.); 2 Shandong Academy of Agricultural Sciences Institute of Agricultural Resources and Environment, Jinan 250132, China; 3 Tianjin Key Laboratory of Pulp and Paper, College of Light Industry Science and Engineering, Tianjin University of Science and Technology, Tianjin 300457, China; 4 College of Food Science and Bioengineering, Tianjin Agricultural University, Tianjin 300392, China

**Keywords:** steam explosion, humification, vegetable wastes, structural characteristics, humic acids, hydrothermal treatment

## Abstract

In this work, steam explosion (SE) was exploited as a potential hydrothermal-humification process of vegetable wastes to deconstruct their structure and accelerate their decomposition to prepare humified substances. Results indicated that the SE process led to the removal of hemicellulose, re-condensation of lignin, degradation of the cellulosic amorphous region, and the enhancement of thermal stability of broccoli wastes, which provided transformable substrates and a thermal-acidic reaction environment for humification. After SE treatment, total humic substances (HS), humic acids (HA_s_), and fulvic acids (FA_s_) contents of broccoli samples accounted for up to 198.3 g/kg, 42.3 g/kg, and 166.6 g/kg, and their purification were also facilitated. With the increment of SE severity, structural characteristics of HA_s_ presented the loss of aliphatic compounds, carbohydrates, and carboxylic acids and the enrichment of aromatic structures and N-containing groups. Lignin substructures were proved to be the predominant aromatic structures and gluconoxylans were the main carbohydrates associated with lignin in HA_s_, both of their signals were enhanced by SE. Above results suggested that SE could promote the decomposition of easily biodegradable matters and further polycondensation, aromatization, and nitrogen-fixation reactions during humification, which were conducive to the formation of HA_s_.

## 1. Introduction

Vegetable wastes mainly refer to the parts of vegetables that are unusable or discarded throughout the food supply chain, from initial agricultural production down to final household consumption, including the whole plants and their roots, stems, leaves, flowers, and fruits, etc. [1,2]. With the improvement of people’s living standards, the demand for vegetables in quantity and quality has gradually gone up, resulting in an increased production amount of vegetable wastes. According to statistics, over 50% of vegetable wastes are lost or wasted during production, distribution, and consumption [1,3]. Conventional treatments and disposals for vegetable wastes are: deep burying and dumping, composting to produce fertilizer, fermenting to prepare silage, feeding earthworms, livestock, etc. [2,3]. Due to the typical characteristics of vegetable wastes such as large quantity and variety, high humidity and perishability, strong harvesting dependence on seasons, etc., there are managerial and technical constraints in the utilization process of vegetable wastes, which restrict the application and popularization of the above methods and make the final disposal rate of vegetable wastes less than 20% [4]. For instance, the low processing efficiency of vegetable wastes for organic fertilizer is attributed to the slow decomposing speed, poor composting maturity, and land-occupation problem of existing composting techniques [2]. They inevitably hinder subsequent agricultural operations and result in the infestation and transmission risks of pests and diseases, so that farmers prefer to bury the vegetable wastes far away. Hence, the large number of vegetable wastes generated from municipal and agro-industrial sectors exhibited a significant global environmental management and disposal issue, prominently restricting the green development of the vegetable industry. Thus, identifying simple technologies for fast reduction and efficient utilization of vegetable wastes with low operation costing and non-polluting impact is a high priority.

Hydrothermal treatment is one of the practical and versatile ways to achieve the effective conversion of biomass. It can achieve the purpose of carbon sequestration and biomass energy utilization, which has received extensive research broadly [5,6]. SE is a typical thermo-chemical process, in which water acts as the reaction medium and catalyst to cause the complex chemical reactions under a certain pressure, thereby cutting off the long chains of biomacromolecules and eliminating the harmful substances and odors [7,8]. The organic carbon stored in biomass could be transferred to the stabilized humus-like substance for rapid storage (generally no more than 2 h) during this process. In fact, because of its relatively stable structure and complex chemical components, it is difficult for biomass to form carbon microspheres through hydrothermal treatment without adding any catalyst. While, if the reaction temperature is higher and the duration time is longer, the orderly crystalline structure can be destructed and reconstructed with the formation of humified fractions and the darkening of the color [6]. There are a series of hydrolysis and polymerization reactions that occurred under high-pressure hydrothermal conditions [2,5,9]. The major structural components in biomass would be hydrolyzed to small molecules, such as monosaccharides and oligosaccharides, amino acids and short-chain peptides, polyphenols, and organic acids, etc. Then they were further decomposed, for example, xylose and arabinose were decomposed to furfural, and glucose was degraded into 5-hydroxymethylfurfural (5-HMF), levulinic acid, or γ-valerolactone [7,8]. These products contained active groups (like carboxyl, hydroxyl, aldehyde, ketone, etc.) and participated in some condensation reactions under a thermal-acidic environment, thereby having opportunities to transform into stable humic substances. Besides, the steaming stage has a uniform sterilization effect on vegetable wastes, during which some harmful microorganisms attached to the vegetable surface or between the damaged tissues would be killed by the thermal action [10]. Moreover, the instantaneous decompression during the explosion stage would cause the adiabatic expansion of pressurized steam and the flash evaporation of liquid water in plant tissues, resulting in the physical-tearing effects of plant fibers in order to facilitate the dissolution of humic substances [2,7]. Thus, SE represents a suitable hydrothermal treatment for promoting the humification of organic matters in vegetable wastes, thus improving their agronomic value and minimizing possible negative environmental impact. At present, the SE apparatus has been successfully scaled up from laboratory scale to industrial scale (up to 50 m^3^) [7]. As compared with other hydrothermal technologies, SE offers several attractive features, including lower environmental impact without adding chemical catalysts and discharging three wastes, lower equipment investment and energy consumption, and better feasibility at industrial scale development [7,8].

Thus, the aim of this study is to exploit a potential hydrothermal-humification technique of vegetable wastes. Taking the wasted broccoli stems as a studied object, hydrothermal deconstruction of broccoli wastes was comparatively investigated on the morphological structure, chemical composition, and thermodynamic property by SE treatment. The formation of humic substances and their distribution variation under different SE conditions were studied and the structural characteristics of humic acids were performed using advanced methods including elemental analysis, Fourier transform infrared (FTIR) spectroscopy, X-ray photoelectron spectroscopy (XPS), and two-dimensional ^13^C–^1^H HSQC-nuclear magnetic resonance (2D-NMR). This work allows us to understand the hydrothermal humification and deconstruction effects of SE technology and thus offers new insights to apply SE technology in the disposal of abundant vegetable wastes. It also has positive significance for the efficient preparation and product development of humic substances from vegetable wastes.

## 2. Results and Discussion

### 2.1. Effects of Steam Explosion on Physicochemical Properties of Broccoli Waste

#### 2.1.1. Morphological Structure

**Figure 1:**
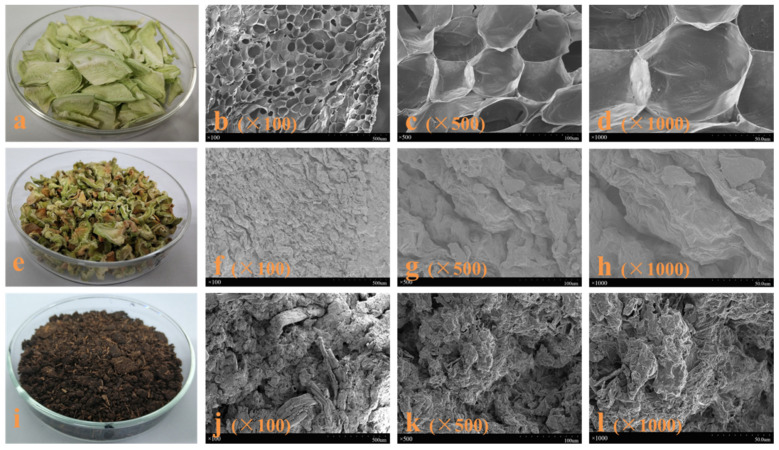
Optical and SEM images of freeze-dried broccoli sample (**a**–**d**), oven-dried broccoli sample (**e**–**h**), and SE broccoli sample at 204 °C for 10 min (**i**–**l**).

The morphological features of broccoli samples after freeze-drying, oven drying, and SE treatment (at 204 °C for 10 min) were observed through optical and SEM imaging as shown in Figure 1. For the freeze-dried sample (Figure 1a–d), its original tissue, cell, and cell wall structures remained almost unchanged with a smooth and intact surface. Once exposed to a thermal environment, the tissue and cell surface of the oven-dried sample (Figure 1e–h) was shrunk and curly with a dark edge due to dehydration. After SE, the particle size, color, and microstructure of the broccoli sample (Figure 1i–l) have undergone great changes. The sample was exploded to fibrous fractures and tended to clump together, some parts even became slurry. The fiber bundles and parenchyma cells of the SE sample were destroyed and presented a disorganized, rugged, and rough morphology after treated at 204 °C for 10 min. Besides, SE deepened the darkness of the broccoli sample, changing its color from green to brown. It could be related to the successive degradation of carbohydrate structures to acetic, formic acids, and other pyroligneous acids, as well as the polymerization of furans (or aromatic rings) and other sugar degradation products, which ultimately led to the production of char at severe SE conditions [8,11]. Apparently, the degree of above destruction increased with the increment of steaming temperature and time. It could be elucidated by the coupling effect of the hydrolytic chemical reactions and intense shearing forces of the SE process. During the steam cooking stage, the hydrothermal atmosphere caused a series of Maillard reactions to induce non-enzymatic browning, and also played a role in softening the fibrous structure [8,11]. Then during the explosion stage, instantaneous decompression action made superheated water flash into steam, and the steam volume abruptly expanded, thereby destroying the porous structure of the broccoli sample at multi scales [7,9].

#### 2.1.2. Chemical Composition

The chemical composition of broccoli samples before and after SE treatment was investigated as shown in Table 1. Notably, water extracts accounted for a large proportion of chemical composition (exceeding 50 wt.%). Its content is firstly increased and then decreased with the increment of steam temperature and retention time. The content of ethanol extracts is also increased by up to 1.47 folds after SE treatment. Much of the literature has confirmed that SE could greatly promote the dissolution and release of soluble substances under certain conditions [8,11,12,13].

The degradation of hemicellulose is one of the major effects of SE conditions tested. Over 60% of hemicellulose fraction was removed from broccoli treated in severer conditions. During the SE process, the high-temperature steam promoted the deacetylation of hemicelluloses and the production of acetic acids. The proportion of acetic acids is increased from 0.4 ± 0.0 wt.% for the raw sample to 1.0 ± 0.1 wt.% for the SE sample treated at 220 °C steam temperature for 10 min retention time. Acetic acids in situ catalyzed the partial cleavage of glycosidic bonds in the hemicellulose backbone and *β*-O-4′ aryl-ether bonds in lignin [7,8]. Hemicelluloses were thus easily hydrolyzed to oligo- and mono-saccharides by the released organic acids, and mono-saccharides were further degraded to furfural and 5-HMF [11]. Additionally, water in substrate could act as an acid at high temperature and also contribute to the formation of thermal-acidic conditions, prompting the auto-hydrolysis of hemicelluloses and the formation of decomposition products [8,11].

The AIL proportion showed a noticeable increase after SE treatment, which was the other major effect induced by this hydrothermal process. It was increased by around 3~5 folds after SE treatment. The formation of extraneous polymeric lignin-like materials (“pseudolignin”) by condensation reactions of carbohydrate and lignin degradation products, particularly under high-severity conditions, has been proposed to be responsible for the apparent increase [11,14,15]. The partial repolymerization and relocation of pseudo-lignin on the surface of particles have been observed in SEM images of other feedstocks [16]. In contrast, there was a slight decline in ASL percentage. It has been confirmed that the humic substances in mature compost are mainly formed through complex interactions of lignin, polysaccharides, and nitrogen-containing compounds, with more aromatic compounds, more hydroxyl groups, and fewer carbohydrates [17,18]. The depolymerized products of lignin contained many aromatic and aliphatic hydroxyl groups, which played an important role in the formation of humic substances.

As for cellulose, it presents a little increment after SE treatment, which is presumed to be a relatively proportional change, while the measured value is, in fact, reduced. According to previous studies, the amorphous cellulose could be released and degraded into oligosaccharides or monosaccharides by SE and the microcrystalline structure would not usually be damaged [11,16]. Under the synergistic effects of high temperature and organic acids, water molecules entered the amorphous and crystalline region of cellulose and caused swelling. The inter-cell and intra-cellular substances were partially dissolved, weakening the firmness of tissues. The glycosidic bonds were broken, and the degree of polymerization was decreased so that the cellulose was gradually decomposed to cellobiose and glucose.

Therefore, the major physicochemical changes of Broccoli samples during the SE process were ascribed to the removal of hemicellulose, the re-condensation of lignin, and the deconstruction of the cellulosic amorphous region. It removed the structural components and increased the content of non-structural components, both of which might increase the chances of conversion to humic substances. Besides, the formation of a thermal-acid atmosphere through the above chemical reactions may exacerbate the formation of humic substances to some extent.

#### 2.1.3. Thermodynamic Property

**Figure 2:**
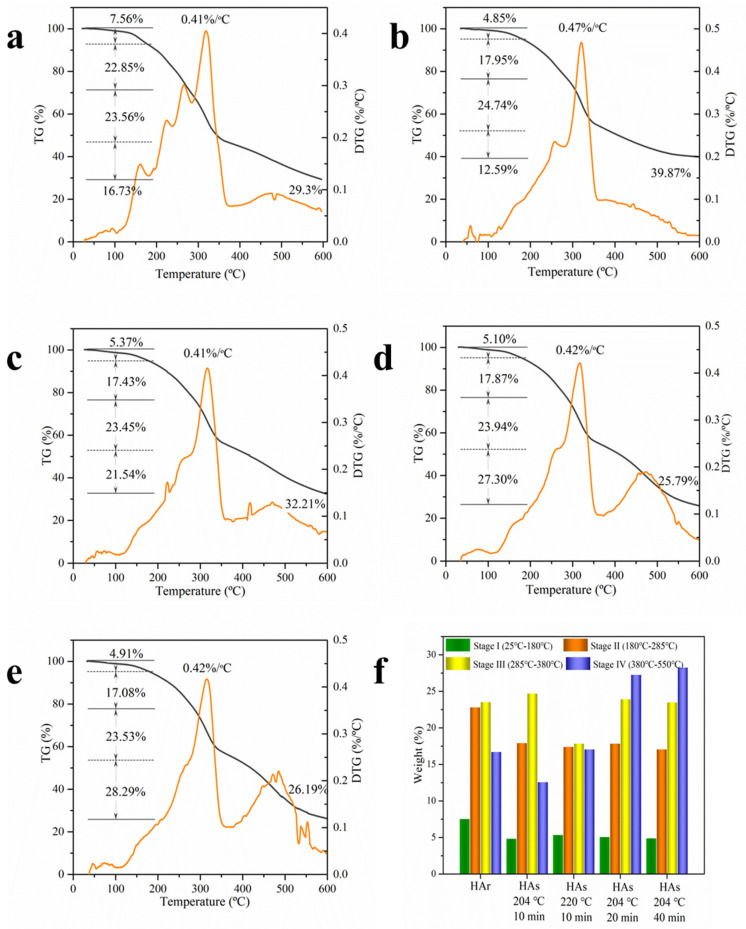
TG and DTG curves of raw broccoli (**a**) and SE broccoli (**b**) 184 °C 10 min; (**c**) 204 °C 10 min; (**d**) 220 °C 10 min; (**e**) 204 °C 20 min; (**f**) 220 °C 40 min.

The pyrolysis characteristics and thermogravimetric (TG/DTG) curves of broccoli samples before and after SE treatment are exhibited in Figure 2, respectively. As shown in Figure 2a, the decomposition reaction of the raw sample can be divided into four stages. There is 7.6 wt.% weight loss for the raw sample and 4.9–5.4 wt.% for the treated sample in the first stage from room temperature to 180 °C. The dehydration peak at around 160 °C disappeared with SE treatment. It is attributed to the evaporation of adsorbed water and crystalline water or partial release of some light volatiles in the broccoli stem [19]. The second stage (180–285 °C) is attributed to the thermal depolymerization of hemicelluloses or pectin [20,21]. The maximum temperature and rate of weight loss in this stage gradually decreased from original broccoli (265 °C and 22.9 wt.%) to treated broccoli (258–263 °C and 17.1–18.0 wt.%). The major decomposition occurred in the third stage (285–380 °C), which is attributed to cellulose decomposition [20,21]. The temperature of the maximum degradation rate shifted from 318 °C to 322 °C through SE treatment, respectively. The main reason for this is related to the partial decomposition of amorphous cellulose during the SE process. When the temperature was over 180 °C, fiber began to be thermally degraded rapidly, glucosyl and glycosidic bonds were broken, and some small-molecule gases including carbon monoxide, CO_2_, and methane were released [19]. The TG curve decreases rapidly, and the weight loss rate increases gradually. CO_2_ originated from the cleavage of the terminal group of acetyl and carboxyl groups in the hemicellulose and cellulose, and the cleavage of lateral chain carboxyl groups and carbonyl groups and esters in the lignin [19]. However, because of having an aromatic structure, lignin would undergo combustion at a higher temperature than cellulose or hemicellulose does (which are aliphatic compounds) with a wide temperature range from 250 to 550 °C [20,22]. It can be observed that after being treated, the DTG pattern reveals an appearance of the fourth stage (380–550 °C) corresponding to lignin. This fact suggests the formation of more thermolabile chemical bonds in the lignin fraction by strong thermal decomposition and condensation in the SE process [23]. After, the carbonaceous matters and char of solid residues were continuously decomposed and copolymerized with slow rates until the final temperature, releasing methane and carbon monoxide and so on. At the final temperature, the mass values of pyrolysis residues of raw and SE Broccoli were 29.3 wt.% and 25.8–39.9 wt.%, respectively. After SE, the remaining solid is increased under the weaker processing conditions, which is probably due to enhanced carbonation via repolymerization reaction. Comparing the pyrolysis data between the raw sample and treated samples, SE treatment could increase the thermal degradation rate in the middle and lateral pyrolysis stages and increase the decomposition temperatures slightly. The main reason for this may be related to the partial removal of hemicelluloses and lignin from the broccoli and the higher crystallinity of cellulose. These are consistent with the results obtained in Section 2.1.2.

### 2.2. Humification Phenomenon of Broccoli Waste during Steam Explosion Process

During the hydrothermal environment, broccoli wastes underwent a series of complicated reactions, involving the destruction of organic macromolecules and the compulsive humification to form humic substances. In comparison with raw samples, the values of TOM and HS in SE samples increase from 260.6 ± 1.6 g/kg and 136.4 ± 5.7 g/kg to 371.7 ± 2.3 g/kg and 198.3 ± 2.3 g/kg, respectively, as shown in Table 2. The contents of HA and FA are increased by 131.4% and 41.0%, respectively. The humification rate is the ratio of HA content to FA content, which can indicate the stability of humified products. With the increment of SE severity, the humification rate is gradually improved. Besides, it is found in Table 2 that both reaction temperature and retention time have important impacts on humification reactions.

Humus is a mixture of extraordinarily complex, molecularly flexible organic materials that naturally contains non-identifiable macromolecules. It is usually regarded to be mainly formed by the polymerization of aliphatic and aromatic compounds. Taking saturated steam as the reaction medium and dissolving solvent, complex hydrothermal reactions of degradable organics occurred during the SE process under a certain steam pressure. The harmful substances and unpleasant odor were eliminated, and the long chains of organic macromolecules were cut off, so that the organic carbon stored in biomass could be transferred to the stabilized humus-like substance for rapid storage, resulting in the increase of HS content in SE samples. Humus can be fractionated into specific humic substances mainly including HA and FA [17,18]. FA is majorly composed of polysaccharides (carbohydrates and some alkoxy compounds) and different amounts of alkyl compounds. HA is mainly composed of polysaccharoids, aromatic lignin derivatives, and long-chain alkyl compounds [18]. Its proportion of aromatic structure is larger than that of FA, and it contains polymethylene-rich long-chain aliphatic fragments and lignin fragments that are not easily degraded. The increased content of HA indicated that with the increment of steam temperature and the prolongation of retention time, SE products contained a growing amount of aromatic structure and a certain amount of stable aliphatic structure. This is consistent with the above compositional results of SE broccoli in Section 2.1.2. According to some literature, this trend may be mainly caused by the intermolecular dehydration polymerization of major components in broccoli, the cleavage of aromatic rings in aromatic products, and the re-polymerization of small molecular fragments [24,25,26].

The extraction, separation, and purification methods of humus in broccoli wastes are mainly referred to as the mature technologies in soil research [27,28]. Humus was extracted by NaOH solution and then HA (precipitate) and FA (supernatant) were separated by the acidification with HCl solution. The purification of HA was mainly by XAD-8 resin and then H-saturated cation exchange resin, while the purification of HA was majorly conducted by dialyzing the HA solution with Spectra Por (1000 Dalton MWCO), both of them were finally lyophilized for analysis. The yields of purified HA and FA are shown in Table 2. The yield of purified HA from SE Broccoli is up to 1.5 times higher than that from raw broccoli, and the yield of purified FA is increased by 4.4 times after SE treatment. This phenomenon indicated that HA and FA were enriched in the humified products by SE processing, which effectively prompts the conversion, extraction, and purification of humified fractions.

### 2.3. Structural Characteristics of Humic Acids Originated from Steam Exploded Broccoli Waste

#### 2.3.1. Elemental Composition

The elemental composition and atomic ratios of HA extracted from raw and SE broccoli were listed in Table 3. After SE, the elemental composition of HA_s_ are: C, 46.4–56.4%; H, 6.31−6.93%; O, 30.3−41.7%; N, 3.71−5.36%; S, 0.27–0.52%. The maximum percentages of C, N, and S for HAs increased by 46.63%, 322.05%, and 73.33%, while the minimum percentage of O decreased by 42.85% when compared to HA extracted from raw samples. The C content in HA_s_ is similar to that in HA from soil and lignocellulose but is lower than the content of HA extracted from peat [25,29]. The increasing N content may be attributed to the formation of stable N-rich structures in the hydrothermal-humification process. This result is in agreement with humification theories that hypothesize N incorporation in HS mainly occurring by condensation reactions of proteins and modified lignin and/or other N-containing compounds and quinones derived from lignin [24,30]. The decrease of O content indicates the release of oxygen-containing groups such as carboxyl and hydroxyl from HA_s_ by SE. The increase of S content suggests that SE enhanced the hydrolysis reaction of sulfur-containing compounds in organic matter, thus making more S stabilized in HA_s_.

The H/C ratio can be used to indicate the degree of aromatic condensation and maturity of HA [18,26]. Its value in HA_r_ is 2.09 and decreased to a narrow range from 1.35 to 1.75 in HAs. The H/C ratio values are higher than 1, indicating that an aromatic framework exists in the chemical structures. Some H/C ratios for lignite HA with values greater than 1 have been reported, even though such values are the main characteristics of soil humic acids [18]. These observations are supported by the fact that SE enhanced humification, facilitated the aromatic condensation, and raised the proportion of unsaturated structures to saturated structures. The O/C ratio and N + O/C ratio reflect the degree of oxidation and polarity, respectively [25]. The O/C ratio ranges from 0.32 to 0.54 in lignite HA, as previously reported in the literature, with a typical value of around 0.4 [18]. The values for our samples were within the range and their decrease after SE showed the reduction of HA oxidizability. The N/C ratio in HA_s_ increased significantly, suggesting that SE could concentrate N-rich structures. Higher values of the N/C ratio (frequently around 0.05) are characteristics of humic acids from soil and peat, while lignite humic acids usually exhibit values <0.05 [18].

Based on these interpretations, it can be concluded that SE could promote the decomposition of easily biodegradable matters and further polycondensation and aromatization reactions during HA formation. During the hydrothermal-humification process, the organic matter was first degraded and converted into small molecular substances, and the remaining fraction was then condensed and reorganized to form highly aromatic polymers which were difficult to decompose. The dehydrogenation and the incorporation of N-containing compounds into HA_s_ structures occurred during the SE process. These results are consistent with other humification processes and confirmed by FTIR and 2D-NMR spectra [24,26].

#### 2.3.2. FTIR Spectrometry

**Figure 3:**
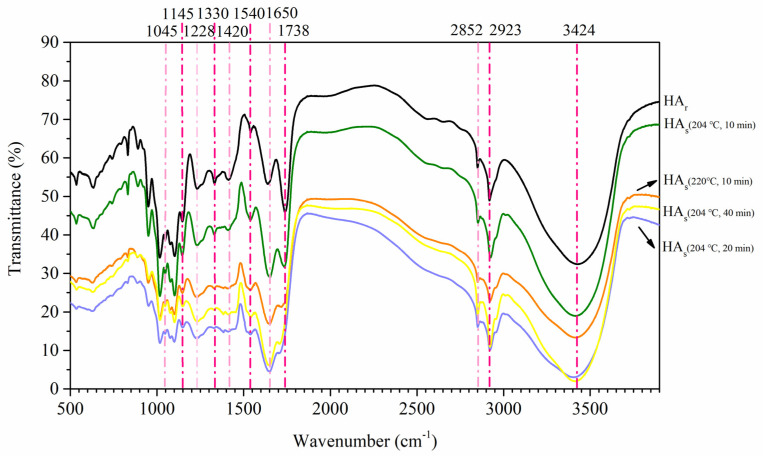
FTIR spectra of HA isolated from raw broccoli and SE broccoli.

The FTIR spectra of HA isolated from treated and untreated broccoli are presented in Figure 3 and their interpretation has been done according to relevant literature [18,31,32,33]. As shown in Figure 3, FTIR peaks displayed a marked difference between the initial humified products and final SE products. The main changes in FTIR spectra of HA fractions from SE broccoli are as below.

(1) The broad band at around 3400 cm^−1^ is attributed to the hydrogen bond-associated-OH stretching or –NH stretching vibration [31,32]. The stretching vibration adsorption bands of aliphatic C-H bonds are in the 3000–2700 cm^−1^ range [31]. The peaks at 2923 cm^−1^ and 2852 cm^−1^ are the asymmetric and symmetric C-H stretching of methyl and methylene groups [18,32]. The intensity of these peaks is weakened after SE treatment, probably because the hydrothermal reactions broke the CH_2_-CH_2_ bonds of the long-chain aliphatic macromolecular compounds, which suggests the reduction of the aliphatic character of HA_s_.
(2) The band at 1710–1740 cm^−1^, ascribed to C=O stretching of carboxyl groups and other carbonyl groups, tends to become less intense with SE treatment [33].
(3) The adsorption band at 1650 cm^−1^ indicates aromatic C=C skeletal vibrations, C=O stretching of quinone and amide groups (amide I band), and C=O of H-bonded conjugated ketones [18,32,33]. This band intensity increased with the SE process extended, which suggests the increased content of aromatic groups.
(4) The peak at 1540 cm^−1^ is associated with stretching vibrations of aromatic C=C and amide C=N (amid II band) and deformation vibrations of N-H [32]. Its higher adsorption of the intensity of HA_s_ suggests it may have a more aromatic ring or amide structures, which is also confirmed by the results of elemental analysis in Section 2.3.1.
(5) The peak at 1420 cm^−1^, which is assigned as the O-H deformation and C-O stretching of phenolic hydroxyl groups, is apparently diminished by the SE treatment [33].
(6) The peak of about 1330 cm^−1^ stands for C-H deformation of CH_2_ and CH_3_ groups, and/or antisymmetric stretching of COO^−^ groups, which appears to decrease after SE treatment [31].
(7) The adsorption intensity of the band at 1228 cm^−1^, which is attributed to the C=O stretching of aryl esters and C-O stretching of aryl ethers and phenols, tends to decrease slightly [33].
(8) The peak of about 1145 cm^−1^ represents aliphatic OH and asymmetric stretching of the C-O-C bridge, and the band around 1030–1080 cm^−1^ stands for C-O stretching of polysaccharides or polysaccharide-like substances [30,31]. The weaker adsorption intensity of these bands indicated that HA_s_ had lower carbohydrate contents than HA_r_.

The above results indicate the progressive degradation of easily decomposable compounds with the relative increase of more complex structures in HA as SE proceeds. Concretely, these observations show the loss of aliphatic materials, carbohydrates, and carboxylic acids and the enrichment of N-containing groups (probably proteinaceous materials) and aromatic structures. The FTIR data further confirm and complement the previous data suggesting that HA_s_ have more aromaticity, polycondensation, and nitrogen-fixation than those noted in the untreated products.

#### 2.3.3. XPS Analysis

**Figure 4:**
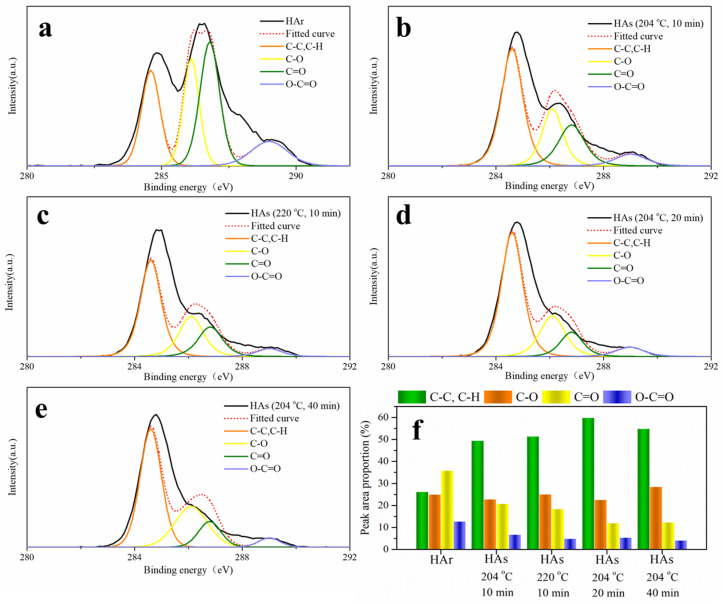
XPS carbon 1s spectra for HAs isolated from raw broccoli and SE broccoli ((**a**): raw sample; (**b**): 204 °C 10 min; (**c**): 220 °C 10 min; (**d**): 204 °C 20 min; (**e**): 204 °C 40 min) and peak area proportion of carbon 1s spectra for HAs (**f**).

XPS spectrometry is a surface analysis technique of supplying chemical composition to a depth of several nanometers under the sample surface [18]. XPS carbon 1s spectra were fitted after spectra deconvolution and four chemical states were distinguished as shown in Figure 4. They indicate four different structural groups with binding energies of about 284.6 eV, 286.1 eV, 286.8 eV, and 289.0 eV corresponding to aliphatic/aromatic carbons C-C, C-H; ether/alcohol carbon C-O; ketonic carbon C=O; and carboxylic carbon O=C-O, respectively [18].

The variation of functional groups in HA_r_ shows that they decrease according to the sequence C=O (35.85%) > C-C, C-H (26.25%) > C-O (25.07%) > O=C-O (12.83%). While after SE, they decrease according to the sequence C-C, C-H (49.52–59.93%) > C-O (22.63–28.55%) > C=O (11.99–20.82%) > O=C-O (4.16–6.77%). As SE severity intensifies, the proportion of C-C, C-H is increased while those of C=O and O=C-O are decreased. The higher proportion of C-C, C-H is probably associated with the more presence of aromatic carbon in HA_s_. The smaller proportion of C=O and O=C-O could refer to the lower amount of carboxyl groups and carbonyl groups in HA_s_. These results are consistent with and complementary to those obtained from elemental analysis and FTIR spectrometry because they reflect a very similar distribution of structural groups in humic acids.

#### 2.3.4. 2D-NMR

**Figure 5:**
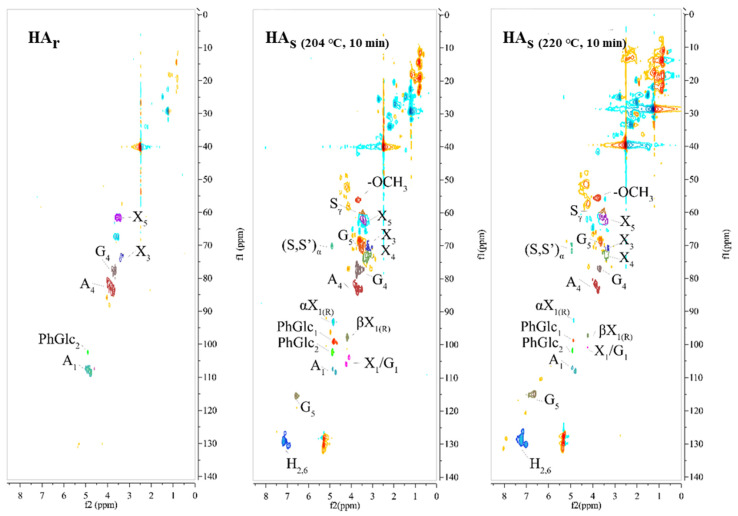
2D HSQC-NMR spectra for HA_s_ isolated from raw broccoli and SE broccoli.

Two-dimensional ^1^H-^13^C HSQC-NMR has been able to provide important structural information and has allowed for the resolution of otherwise overlapping resonances observed in either ^1^H or ^13^C NMR spectra. In the present study, HA samples originated from treated and untreated broccoli were characterized by 2D HSQC-NMR techniques to understand their detailed structures (see Figure 5). HSQC cross-signals of lignin and carbohydrates are assigned by comparison with the published literature, which are listed in Table 4 [34,35,36,37,38]. In general, all the ^13^C NMR spectra of HA could be divided into four chemical regions, which include a series of prominent signals: aliphatic carbons (δ_C_ 0~50), oxygenated aliphatic carbons (δ_C_ 50~100), aromatic carbons (δ_C_ 100~160), and carboxyl/carbonyl carbons (δ_C_ 160~220) [25,32]. And the spectra of ^1^H NMR could be divided into three chemical regions, including aromatic/amide protons (δ_H_ 6~8.5), protons associated with O-alkyl (non-aromatic OCH_n_) groups (δ_H_ 3~5.5), and aliphatic protons (δ_H_ 0.8~3) [25].

As shown in Figure 5, the HA_r_ and HA_s_ spectra exhibit different signals with differences of magnitude, resolution, and chemical shifts. In the aliphatic region, signals (δ_C_/δ_H_ 20~40/1~3) are tentatively assigned to methyl, methylene, and methine groups, which show intensive magnitude in HA_s_ spectra. In the aliphatic region, the C_3_-H_3_ and C_4_-H_4_ correlations from *β*-D-xylopyranoside units (X) are observed at δ_C_/δ_H_ 73.7–75.8/3.22–3.40, and the C_5_-H_5_ correlations from X are observed at δ_C_/δ_H_ 62.4/3.36, which were overlapped with other unassigned cross-signals [34]. The corresponding anomeric correlations of *β*-D-xylopyranoside units (X_1_) are found at δ_C_/δ_H_ 102.1/4.25, whereas the minor anomeric correlations of *β*-D-glucopyranoside units (Glc_1_) might be overlapped in this region [37]. According to a previous study, the corresponding anomeric correlations (C_1_-H_1_) of *β*-D-xylopyranoside units acetylated at both C-2 and C-3 (X23) are observed at 98.9/4.71 [38]. The anomeric correlations from the reducing end of (1→4)-*α*-D-xylopyranoside units (*α*X_1_) and (1→4)-*β*-D-xylopyranoside units (*β*X_1_) units are found at δ_C_/δ_H_ 92.8/4.86 and 97.40/4.26, respectively. Additionally, some anomeric correlations from phenyl glycoside linkage units labeled PhGlc_1_ and PhGlc_2_ are observed at δ_C_/δ_H_ 98.4/4.90 and 101.5/4.79, respectively [38]. These results confirm that gluconoxylan is the major carbohydrate associated with lignin macromolecules in HA_s_, and acetyl groups frequently acylate the C2 and C3 positions. Both HA_s_ spectra show prominent signals corresponding to methoxyls (δ_C_/δ_H_ 55.7/3.75) and *β*-O-4′ aryl ether linkages in the side-chain region [37,38] The C*_α_*-H*_α_* correlations in *β*-O-4′ substructures are observed at δ_C_/δ_H_ 71.8/4.86, and the C*_γ_*-H*_γ_* correlations were observed at δ_C_/δ_H_ 59.5~59.7/3.40~3.63 [37,38]. The main cross-signals in the aromatic region of the 2D HSQC spectra of HA_s_ should correspond to the aromatic rings of *p*-hydroxyphenyl (H) and guaiacyl (G) lignin units. The G units show a prominent signal for the C_5_-H_5_ correlations at δ_C_/δ_H_ 114.9/6.64 [38]. The C_2,6_-H_2,6_ aromatic correlation from H units can be clearly detected at δ_C_/δ_H_ 129.0/7.23 [35,38]. These signals indicate that lignin substructures might be the predominant aromatic structures in HA_s_ and their signals are enhanced by SE treatment.

## 3. Materials and Methods

### 3.1. Materials and Reagents

Waste stems of broccoli (*Brassica oleracea* L. var. *italic Planch.*) were obtained from the student canteen of Tianjin University of Science and Technology (Tianjin, China). All standard chemicals and chemical reagents were of analytical grade and purchased from Sigma Chemical Co. (Louis, MO, USA) and Sinopharm Group Chemical Reagent Co., Ltd. (Tianjin, China).

### 3.2. Steam Explosion Process of Broccoli Waste

SE treatment was performed in a 5 L batch vessel (Weifang Derui Biotechnology Co., Ltd., Shandong, China) which was composed of a reaction retort, a receiving tank, and a saturated steam generator. Fresh stems of broccoli were sliced and dried in an oven at 70 °C to reach the moisture content of 40%. Two-hundred-and-fifty-gram samples were top-loaded into the reaction retort and possessed at a certain saturated steam temperature of 184 °C (1.1 MPa), 204 °C (1.7 MPa) and 220 °C (2.3 MPa) for 10 min, and at 204 °C (1.7 MPa) for 10 min, 20 min, and 40 min, respectively. The steaming period terminated with a swift decompression by the ball valve and materials were exploded into the receiving tank. After SE, the samples were dried and stored at room temperature for further use.

### 3.3. Physicochemical Properties Characterization of Steam Exploded Broccoli Waste

The scanning electronic microscopy (SEM) observation of broccoli samples was obtained using a JEOL JSM-6700F system (JEOL, Japan) to get SEM images. Before the measurement, samples were frozen in liquid nitrogen and dried in a vacuum freeze-dryer. Then they were coated with a thin layer of gold using a sputter-coater (Hitachi Science Systems, Japan).

The identified chemical composition, including water extracts, ethanol extracts, cellulose, hemicellulose, ASL, AIL, acetic acid, and ash of raw and SE samples, were determined according to the Laboratory Analytical Procedures of the National Renewable Energy Laboratory (NREL).

The FTIR spectroscopy study of raw and SE samples were measured by IS50 FTIR spectroscopy (Thermo Fisher, Massa, WA, USA). Each sample was prepared according to the potassium bromide technique. The region between 4000 and 500 cm^−1^ was recorded with a resolution of 4 cm^−1^ and 40 scans.

The thermogravimetric analysis (TGA) was performed with a TA instrument (Waters, LLC, New Castle, DE, USA) under a nitrogen flow. About 3 mg sample was placed in an aluminum crucible and heated from 30 °C to 600 °C at a speed of 10 °C/min.

### 3.4. Determination of Humic Substances in Steam Exploded Broccoli Waste

The total organic matter (TOM) contents of raw and SE samples were determined according to NY/T 1121.6-2006. HA content and FA content were measured by sodium pyrophosphate-sodium hydroxide extraction and potassium dichromate oxidation volumetric method according to NY/T 1867-2010, HS content was calculated as the sum of HA content and the FA content.

### 3.5. Isolation and Purification Process of Humified Fractions from Steam Exploded Broccoli Waste

**Figure 6:**
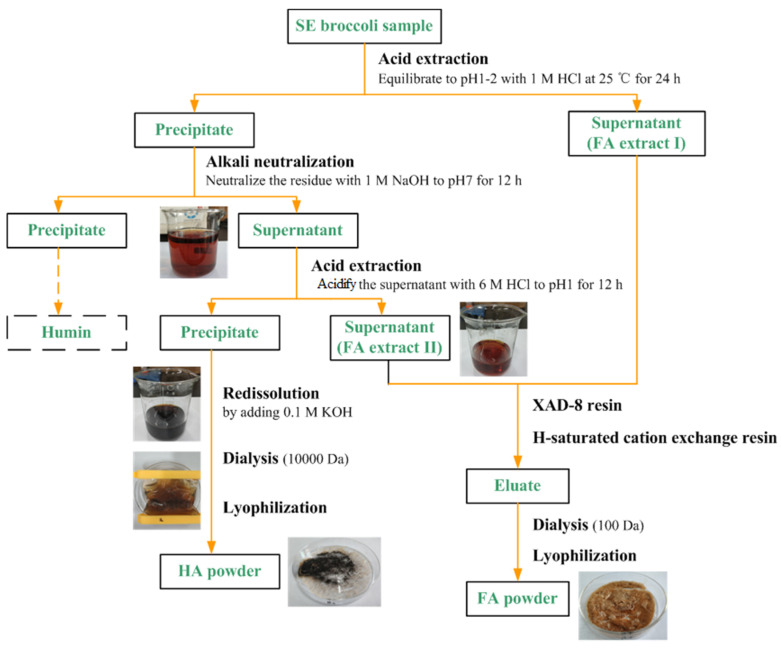
Extraction and purification scheme of humified fractions from steam-exploded broccoli waste.

The isolation and purification of humified fractions were performed according to the International Humic Substance Society (IHSS) recommended method with some modifications (Figure 6). Briefly, HA was precipitated by 6 M HCl solution to adjust leachate pH 1–2 at room temperature and then separated by centrifugation and filtration. Purification and protonation of HA were conducted by dialyzing HA solution (HA redissolved in 0.1 M KOH solution) against DI water, 0.1 M HCl, and DI water sequentially. FA was retained by XAD-8 resin and was eluted out by 0.1 M NaOH solution, which later was allowed to flow through the H-saturated cation exchange resin. Purified HA and FA were freeze-dried for further chemical and spectroscopic analysis. The purified HA from raw and SE samples were regarded as HAr and HAs, respectively.

### 3.6. Structural Characterization of Humic Acids from Steam Exploded Broccoli Waste

Elemental analysis for C, H, O, N, and S of freeze-dried HA samples was performed using an elementary analyzer (Vario EL III, Hanau, Frankfurt, Germany).

FTIR spectroscopy of HA samples was characterized according to the same method as described in Section 2.3.

XPS was obtained by a photoelectron spectrometer (ESCALAB Xi+, ThermoFisher Scientific, Massa, Waltham, MA, USA) with an Al K X-ray source (1486.6 eV). The survey scans were collected using a fixed pass energy of 100 eV and an energy step size of 1.0 eV, whereas the narrow scan had a pass energy of 30 eV and an energy step size of 0.1 eV. The charge effect was corrected by adjusting the binding energy of adventitious C 1s to 284.8 eV.

2D HSQC NMR determination of HA was performed in a Bruker ADVANCE III 400 MHz spectrometer (Bruker Daltonic Inc., Karlsruhe, Germany) with a 4 mm high-resolution liquid probe. HA samples (60 mg) were dissolved in DMSO-*d6* (99.9%, Cambridge Isotopes Laboratories, Tewksbury, MA, USA) to record the 2D HSQC NMR spectra.

### 3.7. Statistical Analysis

All the experiments were performed in triplicate with the average value being reported on a dry basis. The differences between variables were tested for significance using ANOVA and Duncan’s multiple range test. Differences between means were considered significantly different at *p* < 0.05 (SPSS for Window 24.0).

## 4. Conclusions

The impacts of SE on the physicochemical properties of broccoli waste mainly involved the removal of hemicellulose, the re-condensation of lignin, and the degradation of the cellulosic amorphous region, which were accompanied by the porous structural destruction and browning phenomenon. The maximal removal of hemicellulose in the treated materials was 63.77% and the maximal proportion of AIL was 5.1-time higher than that of untreated materials. The maximum pyrolysis temperature and thermal stability of broccoli samples were enhanced by SE treatment. The above changes inevitably provided transformable substrates and assisted in the creation of a thermal-acidic environment for humification reactions. After SE, HS, HA_s_, and FA_s_ contents of broccoli samples accounted for up to 198.3 ± 2.3 g/kg, 42.3 ± 4.5 g/kg, and 166.6 ± 2.0 g/kg on the dry weight and the purification yields of HA_s_ and FA_s_ were also facilitated by up to 1.5 folds and 4.4 folds through SE treatment. With the increment of SE severity, H/C ratio, O/C ratio, and (N + O)/C ratio of HA_s_ decreased but its N/C ratio increased; and the proportion of aliphatic/aromatic carbons C-C/C-H of HA_s_ increased, while its proportions of ketonic carbons C=O and carboxylic carbons O=C-O decreased. The structural characteristics of HA_s_ presented the loss of aliphatic compounds, carbohydrates, and carboxylic acids and the enrichment of aromatic structures and N-containing groups (probably proteinaceous materials). Lignin substructures were proved to be the predominant aromatic structures and gluconoxylans were the major carbohydrates associated with them in HA_s_, and both of their signals were enhanced by SE treatment. The above results indicated that SE could promote the decomposition of easily biodegradable matters and further polycondensation, aromatization, and nitrogen-fixation reactions, which were conducive to the formation of HA_s_. It was concluded that this work provided a potential hydrothermal-humification pretreatment to realize the rapid reduction, innocuity, and utilization of vegetable wastes as well as to prompt the effective preparation and development of biochemical humified products.

## Figures and Tables

**Table 1:** Chemical composition (g/100 g dry weight) of raw broccoli and SE broccoli.

Condition	Water- Extracts	Ethanol- Extracts	Cellulose	Hemicellulose	Acid-Soluble Lignin (ASL)	Acid-Insoluble Lignin (AIL)	Acetic Acid
Raw sample	56.5 ± 2.5	1.8 ± 0.5	9.3 ± 0.6	7.3 ± 0.2	1.4 ± 0.1	2.2 ± 0.3	0.4 ± 0.0
184 °C 10 min	60.4 ± 1.6	2.3 ± 0.6	10.3 ± 0.3	4.2 ± 0.1	0.7 ± 0.1	8.7 ± 0.2	0.4 ± 0.1
204 °C 10 min	61.7 ± 1.9	2.8 ± 0.2	10.5 ± 0.9	3.0 ± 0.2	0.8 ± 0.2	10.2 ± 0.8	0.6 ± 0.0
220 °C 10 min	55.2 ± 0.5	4.2 ± 0.4	11.3 ± 0.2	2.8 ± 0.0	1.1 ± 0.1	12.5 ± 0.0	1.0 ± 0.1
204 °C 10 min	61.7 ± 1.9	2.8 ± 0.2	10.5 ± 0.9	3.0 ± 0.2	0.8 ± 0.2	10.2 ± 0.8	0.4 ± 0.1
204 °C 20 min	53.6 ± 1.5	3.8 ± 0.2	11.2 ± 0.1	3.1 ± 0.2	1.2 ± 0.2	11.9 ± 0.2	0.8 ± 0.0
204 °C 40 min	54.5 ± 0.4	4.5 ± 0.7	11.6 ± 0.6	2.6 ± 0.1	1.0 ± 0.1	13.5 ± 0.2	0.9 ± 0.0

**Table 2:** TOM, HS, HA, and FA content of raw broccoli and SE broccoli.

Condition	Total Organic Matter (TOM) g/kg	Total Humic Substances (HS) g/kg	Humic Acids (HA) g/kg		Fulvic Acids (FA) g/kg		Humification Rate
			Before Purification	After Purification	Before Purification	After Purification	
Raw sample	260.6 ± 1.6	136.4 ± 5.7	18.3 ± 0.5	20.4 ± 2.23	118.1 ± 0.5	6.1 ± 0.03	0.15
184 °C 10 min	322.3 ± 2.8	171.4 ± 0.0	24.8 ± 2.0	30.9 ± 2.0	156.0 ± 4.5	18.8 ± 0.1	0.16
204 °C 10 min	352.7 ± 3.0	183.5 ± 4.5	30.6 ± 3.9	42.4 ± 0.8	163.0 ± 3.9	32.9 ± 0.7	0.19
220 °C 10 min	368.3 ± 4.6	198.3 ± 2.3	42.3 ± 4.5	50.7 ± 0.7	166.6 ± 2.0	28.0 ± 1.5	0.25
204 °C 10 min	352.7 ± 3.0	183.5 ± 4.5	30.6 ± 3.9	42.4 ± 0.8	163.0 ± 3.9	32.9 ± 0.7	0.19
204 °C 20 min	371.7 ± 2.3	185.9 ± 4.3	31.1 ± 6.8	40.7 ± 1.5	151.3 ± 2.7	30.1 ± 0.6	0.21
204 °C 40 min	370.3 ± 1.9	177.5 ± 4.2	34.6 ± 2.7	35.4 ± 1.2	155.0 ± 3.5	27.6 ± 1.3	0.22

**Table 3:** Elemental composition and atomic ratios of HA_S_ extracted from raw broccoli and SE broccoli.

Condition	N %	C %	H %	S %	O %	H/C	O/C	O/H	N/C	N + O/C	Ash/%
Raw sample	1.27	38.4	6.68	0.30	53.0	2.09	1.04	0.50	0.028	1.06	0.39
184 °C 10 min	3.71	46.4	6.75	0.27	41.7	1.75	0.67	0.39	0.068	0.74	1.12
204 °C 10 min	5.36	52.3	6.74	0.51	34.2	1.55	0.49	0.32	0.088	0.58	0.91
220 °C 10 min	5.34	56.4	6.31	0.52	31.1	1.35	0.42	0.31	0.081	0.50	0.40
204 °C 10 min	5.36	52.3	6.74	0.51	34.2	1.55	0.49	0.32	0.088	0.58	0.91
204 °C 20 min	5.14	53.7	6.79	0.48	33.1	1.52	0.46	0.30	0.082	0.54	0.71
204 °C 40 min	5.01	56.3	6.93	0.45	30.3	1.48	0.41	0.27	0.076	0.48	1.04

**Table 4:** Annotation of 2D ^1^H-^13^C HSQC-NMR spectra peaks.

Label	δ_C_/δ_H_	Assignment
-OCH_3_	55.7/3.75	C-H in methoxyls
S_γ_	59.5~59.7/3.40~3.63	C_γ_-H_γ_ in β-O-4′ substructures (S)
X_5_	62.4/3.36	C_5_-H_5_ in β-D-xylopyranoside
(S, S’)_α_	71.8/4.86	Cα-Hα in β-O-4′ substructures (S) and γ-acylated β-O-4′ substructure (S’)
X_3_	73.7/3.22	C_3_-H_3_ in β-D-xylopyranoside
G_5_	73.5/3.60	C_5_-H_5_ in β-D-glucopyranoside
X_4_	75.8/3.40	C_4_-H_4_ in β-D-xylopyranoside
G_4_	76.6/3.60	C_4_-H_4_ in β-D-glucopyranoside
A_4_	82.5/3.75	C_4_-H_4_ in arabopyranose
αX_1(R)_	92.8/4.86	(1→4)-α-D-xylopyranoside (R)
βX_1(R)_	97.4/4.26	(1→4)-β-D-xylopyranoside (R)
PhGlc_1_	98.4/4.90	phenyl glycoside linkages
X23_1_	98.9/4.71	2,3-O-acetyl-β-D-xylopyranoside
X2_1_	100.6/4.73	2-O-acetyl-β-D-xylopyranoside
PhGlc_2_	101.5/4.79	phenyl glycoside linkages
X_1_/G_1_	102.1/4.25	β-D-xylopyranoside/β-D-glucopyranoside
A_1_	109.2/4.90	C_1_-H_1_ in arabopyranose
G_5_	114.9/6.64	C_5_-H_5_ in guaiacyl units (G)
H_2,6_	129.0/7.23	C_2,6_-H_2,6_ in p-hydroxyphenyl units (H)

## Data Availability

The study have provided all the data in the manuscript.

## Sample Availability

Samples of the compounds are not available from the authors.

## Abbreviations

2D-NMR	two-dimentional nuclear magnetic resonance
5-HMF	5-hydroxymethylfurfural
AIL	acid-insoluble lignin
ASL	acid-soluble lignin
FA	fulvic acids
FTIR	Fourier transform infrared
HA	humic acids
HS	total humic substances
SE	steam explosion
TGA	thermogravimetric analysis
XPS	X-ray photoelectron spectroscopy
